# F Plasmids Are the Major Carriers of Antibiotic Resistance Genes in Human-Associated Commensal Escherichia coli

**DOI:** 10.1128/mSphere.00709-20

**Published:** 2020-08-05

**Authors:** Craig Stephens, Tyler Arismendi, Megan Wright, Austin Hartman, Andres Gonzalez, Matthew Gill, Mark Pandori, David Hess

**Affiliations:** a Biology Department, Santa Clara University, Santa Clara, California, USA; b Public Health Program, Santa Clara University, Santa Clara, California, USA; c Alameda County Public Health Laboratory, San Leandro, California, USA; Antimicrobial Development Specialists, LLC

**Keywords:** *Escherichia coli*, F plasmid, antibiotics

## Abstract

The evolution and propagation of antibiotic resistance by bacterial pathogens are significant threats to global public health. Contemporary DNA sequencing tools were applied here to gain insight into carriage of antibiotic resistance genes in Escherichia coli, a ubiquitous commensal bacterium in the gut microbiome in humans and many animals, and a common pathogen. Draft genome sequences generated for a collection of 101 E. coli strains isolated from healthy undergraduate students showed that horizontally acquired antibiotic resistance genes accounted for most resistance phenotypes, the primary exception being resistance to quinolones due to chromosomal mutations. A subset of 29 diverse isolates carrying acquired resistance genes and 21 control isolates lacking such genes were further subjected to long-read DNA sequencing to enable complete or nearly complete genome assembly. Acquired resistance genes primarily resided on F plasmids (101/153 [67%]), with smaller numbers on chromosomes (30/153 [20%]), IncI complex plasmids (15/153 [10%]), and small mobilizable plasmids (5/153 [3%]). Nearly all resistance genes were found in the context of known transposable elements. Very few structurally conserved plasmids with antibiotic resistance genes were identified, with the exception of an ∼90-kb F plasmid in sequence type 1193 (ST1193) isolates that appears to serve as a platform for resistance genes and may have virulence-related functions as well. Carriage of antibiotic resistance genes on transposable elements and mobile plasmids in commensal E. coli renders the resistome highly dynamic.

**IMPORTANCE** Rising antibiotic resistance in human-associated bacterial pathogens is a serious threat to our ability to treat many infectious diseases. It is critical to understand how acquired resistance genes move in and through bacteria associated with humans, particularly for species such as Escherichia coli that are very common in the human gut but can also be dangerous pathogens. This work combined two distinct DNA sequencing approaches to allow us to explore the genomes of E. coli from college students to show that the antibiotic resistance genes these bacteria have acquired are usually carried on a specific type of plasmid that is naturally transferrable to other E. coli, and likely to other related bacteria.

## Abstract (precis)

Rising antibiotic resistance in human-associated bacterial pathogens is a serious threat to our ability to treat many infectious diseases. It is critical to understand how acquired resistance genes move in and through bacteria associated with humans, particularly for species such as Escherichia coli that are very common in the human gut but can also be dangerous pathogens. This work combined two distinct DNA sequencing approaches to allow us to explore the genomes of E. coli from college students to show that the antibiotic resistance genes these bacteria have acquired are usually carried on a specific type of plasmid that is naturally transferrable to other E. coli, and likely to other related bacteria.

## INTRODUCTION

The resistance of pathogenic bacteria to antibiotics is an ongoing threat to global public health (https://www.who.int/antimicrobial-resistance/global-action-plan/en/). The U.S. Centers for Disease Control and Prevention has designated certain antibiotic-resistant *Enterobacteriaceae* as a major public health hazard (https://www.cdc.gov/drugresistance/biggest_threats.html). The most well-known member of this family, Escherichia coli, is ubiquitous as an intestinal commensal in humans, but it can act as a diarrheagenic gastrointestinal tract pathogen (1) or as an extraintestinal pathogen causing urinary tract infections (2) and sepsis (3). Common E. coli lineages causing either intestinal or extraintestinal disease are increasingly found to be resistant to multiple drugs (4–6). Antibiotic resistance in E. coli can arise by mutations in diverse targets or by acquisition of preexisting genes whose products target antibiotics for alteration or efflux (7, 8). Mobile resistance genes have the greatest potential for spread of antimicrobial resistance in the microbiome. The goal of the study presented here was to examine genes underlying antibiotic resistance phenotypes in E. coli by applying genome analysis tools capable of unambiguously assigning the responsible genes to a chromosome or plasmid. Further, we sought to identify the local context of resistance genes to assess their potential for mobility within the genome.

Although genomic analysis of E. coli has largely focused on isolates from pathogenic contexts, deeper analysis of the commensal E. coli population from which such isolates likely emerge will provide new insights into the genetic reservoir that they are drawing from (9–11). Conjugal plasmids are key vectors for disseminating this reservoir of genetic information (12). In commensal E. coli, F plasmids are the most common conjugal plasmids (13), and they were historically the first to be associated with transmissible antibiotic resistance (“R factors”) (14). F plasmids have been prominent in the evolution of medically important lineages such as sequence type 131 (ST131) (15). However, non-F plasmids have also been implicated in the evolutionary dynamics of antibiotic resistance in *Enterobacteriaceae*, such as in recent work on the *mcr-1* gene (encoding colicin resistance) demonstrating that this gene is most often associated with X plasmids (16). Determining the structures of large bacterial plasmids is a significant challenge for DNA sequencing based on short read lengths (17), due to the high frequency of repetitive mobile elements typically residing on them. As a consequence, large plasmids have generally not been carefully analyzed outside of major pathogenic lineages. The advent of low-cost, long-read length sequencing methods is now lowering barriers to such analysis (18). We employed a combination of short-read and nanopore-based long-read sequencing methods to generate complete genome sequences that include all plasmids in complete form, allowing definitive assessment of the genomic context of resistance genes.

## RESULTS

### Isolation and characterization of commensal E. coli.

A collection of 101 commensal E. coli isolates, obtained from healthy college students between 2014 and 2019, were phenotypically characterized for antibiotic resistance. The majority (56/101 [55%]) of the commensal isolates analyzed were phenotypically resistant to at least one of the following classes of antibiotics: β-lactams (36%), sulfonamides (35%), aminoglycosides (34%), trimethoprim (27%), tetracyclines (27%), quinolones (25%), macrolides (17%), or phenicols (3%). Over one-third of the isolates (37%) were multidrug resistant (MDR) (defined as resistant to three or more classes of antibiotics). These 101 isolates were subjected to short-read DNA sequencing to obtain draft-level genome assemblies adequate for resistance gene identification. After alleles were grouped together, 22 distinct acquired resistance genes were identified (Table 1), which accounted for over 85% of observed antibiotic resistance phenotypes. The primary exception was for quinolone resistance (25% of isolates), in which case known mutations in the chromosomal *gyrA* and *parC* genes (19, 20) were present in 23 out of 25 resistant isolates.

**TABLE 1:** Acquired resistance genes identified in commensal E. coli isolates

Drug and resistance gene(s)	No. (%) identified in all isolates (*n* = 101)
β-Lactams	
*bla*_TEM_	31 (31)^*a*^
*bla*_CTX-M_	5 (5)^*b*^
*bla*_SHV-1_	1 (1)
*bla*_CMY_	2 (2)^*c*^
Aminoglycosides	
*strA*, *strB*	27, 27 (27)
*aadA* (3 alleles)	19 (19)^*d*^
*aadB*	1 (1)
*aac(3)-IId*	8 (8)
*aph(3*′)	1 (1)
*sat*	2 (2)
Sulfonamides	
*sul1*	18 (1)
*sul2*	29 (29)
Trimethoprim	
*dfrA* (5 alleles)	27 (27)^*e*^
Tetracyclines	
*tetA*	18 (18)
*tetB*	6 (6)
*tetD*	4 (4)
Macrolides	
*mphA*	17 (17)
*mphB*	1 (1)
Phenicols	
*cmlA*	2 (2)
*cat*	2 (2)
Quinolones	
*qnrS*	1 (1)

a *bla*_TEM-1B_ in 29 isolates, *bla*_TEM-1C_ in 1 isolate, and *bla*_TEM-34_ in 1 isolate.

b *bla*_CTX-M-14_ in 3 isolates and *bla*_CTX-M-27_ in 2 isolates.

c *bla*_CMY-2_ in 1 isolate and *bla*_CMY-M-14_ in 1 isolate.

d *aadA1* in 5 isolates, *aadA2* in 3 isolates, and *aadA5* in 11 isolates.

e *dfrA1* in 4 isolates, *dfrA5* in 5 isolates, *dfrA7* in 2 isolates, *dfrA8* in 1 isolate, *dfrA12* in 3 isolates, and *dfrA17* in 12 isolates.

Commensal E. coli isolates were assessed for phylogenetic diversity by multilocus sequence typing (MLST) inferred from the draft genome assemblies. Among the 59 MLST types identified (data in Tables S1 and S2 in the supplemental material), ST95 (12 isolates), ST69 (8 isolates), and ST10 (7 isolates) were the most abundant. Representatives of all major E. coli phylogroups were present, with B2 constituting the largest set. Isolates from phylogroup D (primarily ST69 and ST38) were notable for a high frequency of multidrug resistance (13/15 isolates [87%]), significantly higher than that of the overall collection (37%, chi-square test, *P* < 0.01).

10.1128/mSphere.00709-20.1 TABLE S1 Genome components for all commensal E. coli isolates completely or near-completely assembled in this work. This table expands on Table 2, showing sizes of all genome components (chromosome and plasmids) for all isolates that were sequenced and fully assembled, as well as all identifiable chromosomal mutations leading to antibiotic resistance. Download Table S1, DOCX file, 0.1 MB. Copyright © 2020 Stephens et al. 2020 Stephens et al. This content is distributed under the terms of the Creative Commons Attribution 4.0 International license.

10.1128/mSphere.00709-20.2 TABLE S2 Additional draft genome assemblies of commensal E. coli analyzed in this work. This table includes data for an additional 51 draft genomes of commensal E. coli isolates that were subjected to short-read (Illumina) sequencing, but which were not subjected to long-read sequencing for complete assembly. Data from these isolates contributed to Table 1. Download Table S2, DOCX file, 0.02 MB. Copyright © 2020 Stephens et al. 2020 Stephens et al. This content is distributed under the terms of the Creative Commons Attribution 4.0 International license.

Acquired antibiotic resistance genes in bacteria are often carried on plasmids, so the presence of known replicons was assessed using PlasmidFinder (21). Based on these replicons, 79% of isolates were predicted to contain at least one large conjugal plasmid, with FIB (66% of isolates) and FII (62%) replicons being most frequent, followed by I-complex replicons (B/O, K, Z, and I1) in 16% of isolates (data in Tables S1 and S2). No other replicons were found in more than one of the draft genomes. The absence of conjugal plasmid replicons in an isolate was associated with pan-susceptibility (19/45 of pan-susceptible isolates lacked identifiable plasmid replicons versus 2/56 of antibiotic-resistant isolates, chi-square test, *P* < 0.001).

In assemblies based on short-read data, contigs containing antibiotic resistance genes, plasmid replicons, or genes encoding components of conjugal machinery were typically short (<20 kb) and linear (17). Limitations to assembly of short-read data were overcome by the addition of long-read sequencing, and integrating both types of read with hybrid assemblers (18, 22, 23). Using either Unicycler or Flye, assembly of a complete chromosome was achieved for 47 isolates, and for another 3 isolates, the chromosome was present in two to four large contigs. These genomes were therefore considered to be fully or nearly fully assembled and sufficient for assignment of antibiotic resistance genes to chromosomes or plasmids. The 50 genomes comprised representatives from six phylogroups (phylogroup A, 4 isolates; phylogroup B1, 6 isolates; phylogroup B2, 25 isolates; phylogroup D, 10 isolates; phylogroup E, 1 isolate; phylogroup F, 4 isolates) and 33 MLST groups and included 29 isolates carrying acquired resistance genes (Table 2), and 21 lacking acquired resistance genes (Table S1).

**TABLE 2:** Plasmids and antibiotic resistance determinants in fully assembled commensal E. coli genomes

Phylogroup	MLST	Isolate	Resistance phenotype(s)^*a*^	Genome component (GenBank accession no.)	Size	Resistance genes (associated mobile elements)^*b*^	Plasmid replicon(s)^*c*^
A	10	SCU-103	AMP, CEF, AZM, STR, SXT, TET	pSCU-103-1 (CP054458)	139 kb	*aadA5*-*dfrA17* (class 1 integron cassette) plus *sul1* adjacent; *bla*_CTX-M-27_ (IS*903C*, IS*Ecp1* flank); *strA-strB* (Tn*5393*') plus *sul2* (Tn*5393*' flank); *mphA* (IS*6100*, IS*26* flank); *tetA* (Tn*1721*')	F1A, F1B, FII
		SCU-118	AMP, STR, SXT, TET	pSCU-118-1 (CP051717)	85 kb	*aadA1-dfrA1* (class 1 integron cassette) plus *sul1* adjacent; *bla*_TEM-1B_ (Tn*2*'); *mphB* (flanked by Tn*402*); *strA-strB* (Tn*5393*') plus *sul2* (RSF1010-like); *tetA* (Tn*1721*')	F1B'
B1	3695	SCU-106	STR, TET	pSCU-106-2 (CP053236)	112 kb	*strA-strB* (Tn*5393*'); *tetA* (Tn*1721* within Tn*5393*')	F1B', FIC(II)
		SCU-308	AMP, STR, SXT	pSCU-308-1 (CP053282)	152 kb	*bla*_TEM-1B_ (Tn*2*') plus *strA-strB* (Tn*5393*') and *sul2*; *dfrA5* (class 1 integron cassette fragment)	F1B', FII
B2	14	SCU-387	AMP, AZM	pSCU-387-2 (CP051690)	39 kb^*d*^	*bla*_TEM-1B_ (Tn*1,2,3-*like'); *mphA*	FII
	73	SCU-112	AMP, CEF (int)	pSCU-112-1 (CP051726)	104 kb	*aadA1'* (class 1 integron cassette fragment); *bla*_SHV-1_ (IS*26* flanking both sides)	F1B', FII, Col156
	91	SCU-121	TET	pSCU-121-1 (CP054329)	68 kb	*tetA* (Tn*1721*')	FII
	95	SCU-108	AMP	pSCU-108-2 (CP051737)	72 kb	*bla*_TEM-1B_ (Tn*2*)	FII
		SCU-123	AMP, STR, SUL	pSCU-123-2 (CP051713)	95 kb	*bla*_TEM-1C_ (Tn*2c*); *strA-strB'* (Tn*5393*') plus *sul2* (Tn*5393*' flank)	B/O/K/Z (B/O)
		SCU-306	AZM, SXT	pSCU-306-1 (CP053232)	129 kb^*d*^	*aadA2*-*dfrA12* (class 1 integron cassette) plus *sul1* adjacent; *mphA* (IS*6100*, IS*26*, 2 copies)	F1B, FII, Col156
	131	SCU-182	AMP, GEN	pSCU-182-1 (CP054376.1, CP054375.1, CP054374.1, CP054373.1)	168 kb^*d*^	*aac(3)-IId* (IS*26*, IS*10*'); *bla*_TEM-1B_ (Tn*2*')	F1A, F1B, FII, Col156
		SCU-481	AMP, AMC, AZM (int), CHL, SXT	pSCU-481-1 (JABLYB000000000.1)	144 kb	*aadB-aacC'-cmlA6* (class 1 integron cassette); *aadA5*-*dfrA17* (class 1 integron cassette) plus *sul1* adjacent; *bla*_TEM-34_ (Tn*2*); *mphA* (IS*26*, IS*6100* flank)	F1A, F1B, FII
	144	SCU-125	STR, SXT	pSCU-125-2 (CP051702)	93 kb	*dfrA5* (class 1 integron cassette) plus *sul1* adjacent; *strA-strB* (Tn*5393*', IS*CR2*') plus *sul2* (Tn*5393*' flank)	B/O/K/Z (Z)
	357	SCU-124	AMP	pSCU-124-2 (CP051708)	73 kb	*bla*_TEM-1B_ (Tn*2*)	FII
	1193	SCU-147	AMP, AZM, GEN, STR, SXT, TET	pSCU-147-1 (CP054326)	105 kb	*aac(3)-IId* (IS*10*', IS*26* flank); *aadA5-dfrA17* (class 1 integron cassette fragment); *bla*_TEM-1B_ (Tn*1,2,3-*like');| *mphA* (IS*6100*, IS*26* flank); *strA-strB* (Tn*5393*') plus *sul2* (Tn*5393*' flank); *tetA* (Tn*1721*')	F1A, F1B, Col156
		SCU-204	STR, SUL	pSCU-204-1 (CP054414.1)	88 kb	*strA-strB* (Tn*5393*') plus *sul2* (RSF1010-like)	F1A, F1B, Col156
		SCU-390	AMP, STR, SUL	pSCU-390-1 (CP054321)	91 kb	*bla*_TEM-1B_ (Tn*2*'); *strA-strB* (Tn*5393*') plus *sul2* (RSF1010-like)	F1A, F1B', Col156
	2279	SCU-479	AMP, AMC, CEF, CHL, STR, SUL, TET	Chromosome (CP054317)	5.2 Mb	*bla*_CTX-M-14_ (IS*Ecp1*); *bla*_CMY-121_ (IS*Ecp1*); *bla*_TEM-1B _ (IS*Ecp1*); *strA-strB* (Tn*5393*') plus *sul2* (Tn*5393*' flank); *tetA* (Tn*1721*')	
D	38	SCU-164	SXT, TET	Chromosome (CP054343)	5.4 Mb^*d*^	*dfrA7* (class 1 integron cassette) plus *sul1* adjacent; *sul2* (IS*5075*, IS*CR2*); *tetD* (flanked by IS*26* and Tn*2*')	
		SCU-397	AMP, CEF, CHL, STR, SXT, TET	Chromosome (CP054828.1)	5.3 Mb^*d*^	*bla*_CTX-M-14_ (2 copies, each between IS*ECP1* and IS*903B*'); *bla*_TEM-1B_ (Tn*2*'); *dfrA7* (class 1 integron cassette) plus *sul1* adjacent; *strA-strB* (Tn*5393*') plus *sul2* (Tn*5393*' flank); *tetD* (IS*26*, Tn*2*' flank); *catA1*	
		SCU-486	AMP, CEF, AZM, GEN, STR, SXT, TET	Chromosome (CP051749)	5.2 Mb	*bla*_CTX-M-14_ (IS*903B'*, IS*Ecp1* flank); *strA-strB* (Tn*5393*'); *sul2* (IS*5075*, IS*CR2* flank); *tetD* (IS*26*, Tn*2'* flank); *bla*_TEM-1B_ (Tn*2'*); *catA1* (IS*26* flank)	
				pSCU-486-1 (CP051750)	84 kb	*aac(3)-IId* (IS*26*, IS*Kpn11-like*' flank); *bla*_CTX-M-14 _ (IS*903B*', IS*Ecp1* flank); *dfrA5* (class 1 integron cassette) plus *sul1* adjacent; *mphA* (IS*6100*, IS*26* flank)	F1B', FII
	69	SCU-313	AMP, AZM, GEN, STR, SUL, TET	pSCU-313-1 (CP051695)	105 kb	*aac(3)-IId* (IS*26*, IS*10*'); *aadA5-dfrA17* (class 1 integron cassette) plus *sul1* adjacent; *bla*_TEM-1B _ (Tn*1,2,3-*like'); *mphA* (IS*26*, IS*6100*); *strA-strB* (Tn*5393*') plus sul2 (Tn*5393*' flank, IS*26*); *tetA* (Tn*1721*')	F1A, F1B'
		SCU-482	AMP, AZM, STR, SXT	pSCU-482-1 (CP053248)	145 kb	*aadA5-dfrA17* (class 1 integron cassette) plus *sul1* adjacent; *bla*_TEM-1B_ (Tn*2*'); *mphA* (IS*26*, IS*6100*' flank); *strA-strB* (Tn*5393*') plus *sul2* (RSF1010-like)	F1B', FII, Col156
	106	SCU-318	AMP, STR, SUL, TET	pSCU-318-1 (CP051693)	105 kb	*bla*_TEM-1B_ (Tn*2*'); *strA-strB* (Tn*5393*') plus *sul2* (Tn*5393'* flank); *tetB* (Tn*10*')	F1B, FII
	394	SCU-105	AMP, CEF, AZM, STR, SXT	Chromosome (CP051738)	5.2 Mb	*dfrA1-sat2-aadA1* (class 2 integron cassettes in Tn*7*)	
				pSCU-105-1 (CP051739)	173 kb^*d*^	*strA-strB* (Tn*5393*'); *sul2* (IC*R2*', IS*5075*' flank)	F1B, FII
				pSCU-105-2 (CP051740)	9.7 kb	*bla*_TEM-1B_ (Tn*2*'); *mphA* (IS*6100*', IS*26*' flank)	
	963	SCU-109	AMP, AMC, CEF, GEN	Chromosome (CP051733)	5.0 Mb	*bla*_CMY-2_	
				pSCU-109-1 (CP051734)	110 kb	*aac(3)-IId* (IS*26*, IS*10*' flank); *bla*_TEM-1B_ (Tn*2*')	F1B', FII, Col156
F	62	SCU-175	AMP, AZM, STR, SXT, TET	pSCU-175-1 (CP054380.1)	124 kb	*aadA1-dfrA1-sat2* (class 2 integron in Tn*7*); *mphA* (IS*26*, IS*6100* flank); *sul2* (IS*CR2*' flank); *tetB* (Tn*10*')	B/O/K/Z (Z)
				pSCU-175-2 (CP054381.1)	72 kb	*bla*_TEM-1B_ (Tn*2*)	FII
	379	SCU-172	AMP	pSCU-172-3 (CP054356)	76 kb	*bla*_TEM-1B_ (Tn*2*)	FII
	648	SCU-120	AMP (int), CEF, AZM, STR, SXT, TET	pSCU-120-1 (CP054336)	143 kb	*aadA5-dfrA17* (class 1 integron cassette) plus *sul1* adjacent; *mphA* (IS*6100*, IS*26* flank); *tetA* (Tn*1721*')	F1A, F1B', FII
				pSCU-120-3 (CP054338)	6.2 kb	*strA-strB* (Tn5*393*') plus *sul2* (Tn*5393*' flank)	

a Abbreviations: AMP, ampicillin; AMC, amoxicillin-clavulanic acid; AZM, azithromycin; CEF, cephalothin; CHL, chloramphenicol; GEN, gentamicin; KAN, kanamycin; NAL, nalidixic acid; NOR, norfloxacin; STR, streptomycin; SUL, sulfamethoxazole alone; SXT, sulfamethoxazole-trimethoprim; TET, tetracycline; TMP, trimethoprim alone. “int” in parentheses indicates that the size of the zone of inhibition for the antibiotic met the manufacturer’s criteria for “intermediate” resistance. Note that quinolone resistance is reported here only when due to an acquired gene; resistance due to chromosomal mutations is reported in Table S1 in the supplemental material.

b Identification of antibiotic resistance genes was done with ResFinder (48). A prime symbol indicates that the identified antibiotic resistance gene was incomplete (between 60 and 90% present). Mobile elements were identified using the Galileo Antimicrobial Resistance (GAMR) software (24). A prime symbol indicates that the transposable element was smaller than the published full version of the element. “flank” indicates that the resistance gene was not within the identified mobile element, but within 1 kb adjacent to it.

c Identification of plasmid replicons was done with PlasmidFinder (21). A prime symbol indicates that the identified replicon sequence was incomplete (between 60 and 90% present).

d Assembly was noncircular, suggesting gap of unknown size between ends.

### Local context of antibiotic resistance genes.

To better understand how acquired antibiotic resistance genes are mobilized in commensal E. coli, these genes in the completely assembled genomes were examined for surrounding mobile genetic elements such as insertion sequences (ISs), transposons (Tns), and integrons (Table 2) (24, 25). *bla*_TEM-1_ was always found in a Tn*2* transposable element, though a minority (6/18) resided in a full-length Tn*2* (∼5 kb). In the majority of the partial Tn*2* elements, much of the Tn*2* sequence upstream of *bla*_TEM-1_ was replaced by IS*26*, reducing it to 1.2 to 1.6 kb. In these cases, a second IS element (*1A* or *CR2*) was located on the other flank of the partial Tn*2*. *tetA* was always found on Tn*1721*, and *tetB* on some form of Tn*10*. *strA* and *strB* were always located on Tn*5393*, usually with *sul2* immediately adjacent, followed by IS*26*, suggesting that this entire set moves as a unit. As with Tn*2*, only a few isolates carried complete versions of Tn*1721* or Tn*5393*, with the sizes of the residual elements varying. In four isolates, *sul2* was located in the context of IS*5075*/IS*CR2*, rather than adjacent to Tn*5393*. *mphA* was always found as part of the mobile three-gene cluster between IS*26* and IS*6100* fragments.

**FIG 1:**
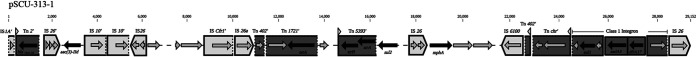
Conserved cluster of antibiotic resistance genes, transposable elements, and a class I integron in pSCU-313-1. Transposable elements and resistance genes were identified using ResFinder (48) and GAMR (24) and visualized using BioRender. IS elements are indicated by light gray boxes, with their name above the box; transposons and the class 1 integron are indicated by dark gray boxes, with their name above. Conserved inverted repeats known to be associated with transposable element boundaries are indicated by triangles above the boundaries. Dashed lines indicate breakpoints (defined by sequence alignment) of interrupted elements; partial elements are indicated by a prime symbol following their name. Antibiotic resistance genes are indicated by black arrows, with their name underneath.

Eleven (38%) of the 29 isolates in Table 2 contained intact class 1 integrons (26) carrying one to three resistance genes, and three contained partial class 1 integrons. Of the 14 class 1 integrons observed, only two were located on chromosomes (SCU-164 and SCU-397). Twenty-five intact resistance genes were found as cassettes in class 1 integrons (intact or partial). Figure 1 shows the most common cassette configuration, with *dfrA17* (trimethoprim resistance) and *aadA5* (aminoglycoside resistance). Alleles of *dfrA* and *aadA* were found as cassettes; the only other intact cassette was *cmlA* (chloramphenicol resistance). *sul1* was present adjacent to the cassette regions of 11 intact class 1 integrons, but it was absent in three partial integrons and present in one partial integron lacking the cassette region. One isolate (SCU-105) contained an intact Tn*7*-associated class 2 integron on the chromosome with *dfrA1*, *satA1*, and *aadA1* cassettes. A second isolate (SCU-175) contained a partial class 2 integron with only fragments of Tn*7* in the adjacent sequence. *aadA* and *dfrA* genes were found only in the context of class 1 and 2 integrons. In total, 42 acquired resistance genes (27% of the total) were associated with class 1 or 2 integrons.

### Plasmids and resistance genes.

**FIG 2:**
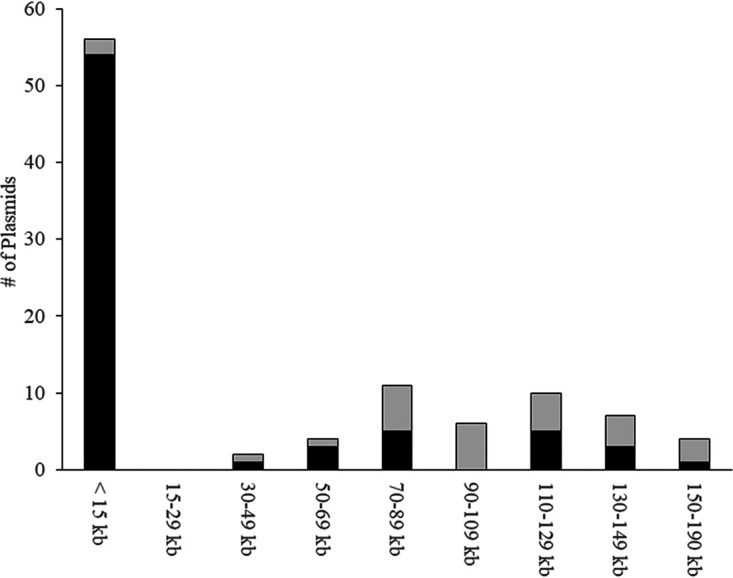
Acquired antibiotic resistance genes are primarily on large (>70-kb) plasmids in commensal E. coli. The *y* axis indicates the number of plasmids identified. Total (black and gray) bars indicate all plasmids in each size range from the genomes of antibiotic-resistant isolates (Table 2); black bars indicate only the plasmids that actually contained acquired antibiotic resistance genes.

In the fully assembled genomes, plasmids partitioned into two general pools, designated here as “small” (1 to 13 kb; *n* = 86; mean size,4.6 kb) and “large” plasmids (26 to 190 kb; *n* = 63; mean size, 103 kb). Figure 2 shows the size distribution of plasmids from the subset of antibiotic-resistant isolates (gray bars); the size distribution of plasmids from antibiotic-susceptible isolates was similar. The majority of the 63 large plasmids were associated with F replicons (49/63 [77%]), and in most cases, multiple subtypes of F replicons were found on the same plasmids. Ten plasmids had IncI complex replicons (Z, B/O, K, or I1). F- and I-complex replicons are typically associated with plasmids capable of conjugation, and the genes encoding components of the conjugal machinery typically take up 35 to 40 kb for both types of plasmids, although many of the F plasmids were missing at least 20% of the conjugation-associated genes (data not shown). None of the assembled plasmids contained replicons of multiple types, and only two putative plasmids had no identifiable replicons using PlasmidFinder (21). Plasmids encoding one or more antibiotic resistance genes were primarily from the large plasmid pool (Fig. 2), with two exceptions discussed more below. The fully assembled genomes from isolates containing acquired resistance genes (*n* = 29 isolates) had significantly more large plasmids per isolate (1.6 ± 0.6) than the genomes from isolates lacking acquired resistance genes (*n* = 21 isolates, 0.95 ± 0.79 large plasmids/isolate) [independent *t* test, *t*(48) = 2.78, *P* = 0.0077].

Nearly 80% (123/154 [78%]) of acquired antibiotic resistance genes in the fully assembled commensal E. coli genomes resided on plasmids (Table 2). Most plasmid-borne resistance genes (103/123 [84%]) were on molecules containing at least one F replicon. Fifteen of the remainder were on three IncI complex plasmids in isolates SCU-123 (B/O replicon), SCU-125 (Z replicon), and SCU-175 (Z replicon). Finally, five were located on two small plasmids (pSCU-105-2 and pSCU-120-3). Only six isolates carried acquired resistance genes on their chromosome (Table 2), totaling 30 acquired resistance genes. The validity of assignment of resistance genes to plasmids or chromosomes was confirmed by electroporation of purified genomic DNA preparations into laboratory E. coli; predicted chromosomal loci never generated antibiotic-resistant electroporants (data not shown).

**FIG 3:**
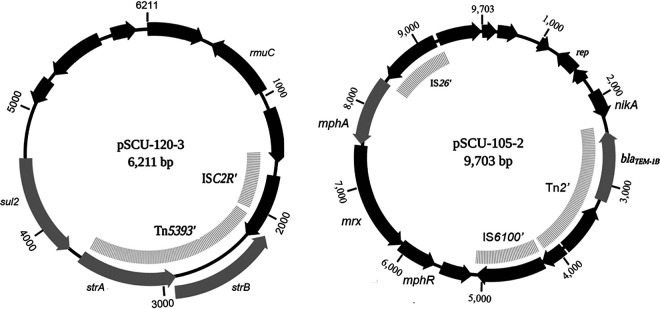
Rare small plasmids from commensal E. coli containing antibiotic resistance genes. Transposable elements and resistance genes were identified using ResFinder (48) and GAMR (24) and visualized using BioRender.

Small plasmids rarely contained antibiotic resistance genes, but there were two exceptions. pSCU-105-2 is a 9.7-kb plasmid containing *bla*_TEM-1_ and *mphA* (macrolide resistance), and pSCU-120-3 is a 6.2-kb plasmid containing *strA-strB* (streptomycin resistance) and *sul2* (sulfonamide resistance) (Fig. 3). Based on read coverage data, pSCU-105-2 was present at roughly 50 copies per chromosome equivalent, and pSCU-120-3 was present at ∼8 copies per chromosome. These plasmids were readily transferred by electroporation into laboratory E. coli strains and stably maintained (data not shown). pSCU-105-2 is a ColE1-type plasmid in which the colicin E1 gene and associated immunity function have been replaced by a 6-kb mobile element comprised of Tn*2* containing *bla*_TEM-1_ and a macrolide resistance locus (*mphA-mrx-mphR*). The pSCU-120-3 replication functions appear to reside in a 3-kb backbone found in several other plasmids (pSCU-103-4, pSCU-105-4, and pSCU-175-5), to which a 3-kb composite element is attached containing a partial Tn*5393* (*strA-strB*) followed by *sul2*.

Multidrug-resistant isolates often exhibited regions in which multiple genetic elements (ISs, Tns, and/or integrons) aggregated into larger, potentially mobile units (27, 28). The largest conserved resistance locus found in the 29 fully assembled isolates was a 19-kb segment shared by plasmids in isolates SCU-103, SCU-147, and SCU-313 (Fig. 1). These plasmids (pSCU-103-1, pSCU-147-1, and pSCU-313-1) are otherwise not closely related, sharing only 50 to 60% of their contents, nor are the host E. coli closely related, coming from distinct MLSTs and phylogroups.

**FIG 4:**
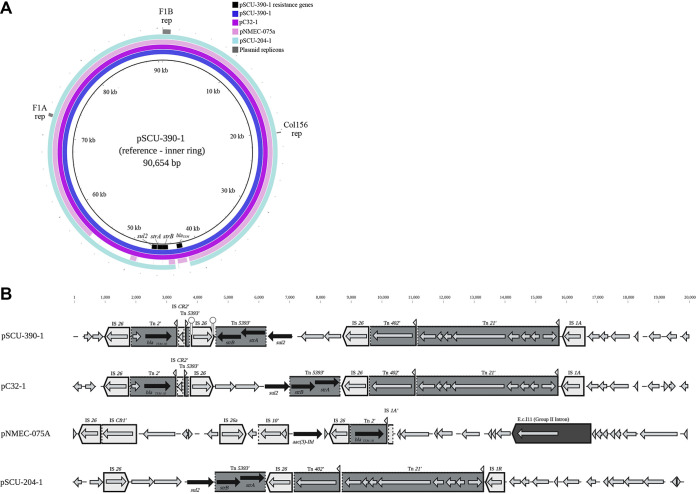
A large conserved plasmid carrying antibiotic resistance is found in ST1193 isolates. (A) Alignment of plasmids pSCU-390-1, pSCU-204-1, pNMEC-075A (GenBank accession no. CP030112.1), and pC32_1 (GenBank accession no. CP041619.1) using BRIG (BLAST Ring Image Generator) (50); numbering starts at the F1B replicon. Select genetic regions shared by all of the plasmids are indicated on the outside ring, including plasmid replicons identified by Plasmid Finder. Antibiotic resistance genes in plasmid pSCU-390-1 are annotated in the inner ring. The variable region from approximately 40 to 60 kb, indicated by gaps in the alignment, is shown in panel B. (B) Comparison of the variable regions located between 40 and 60 kb in the conserved ST1193 plasmids. Transposable elements and resistance genes were identified using ResFinder (48) and GAMR (24) and visualized using BioRender. IS elements are indicated by light gray boxes, with their name above the box; transposons and the class 1 integron are indicated by dark gray boxes, with their name above. Conserved inverted repeats known to be associated with transposable element boundaries are indicated by triangles above the boundaries. Dashed lines indicate breakpoints (defined by sequence alignment) of interrupted elements; partial elements are indicated by a prime symbol following their name. Antibiotic resistance genes are indicated by black arrows, with their name underneath.

Only one complete plasmid with antibiotic resistance genes was found to be highly conserved in multiple isolates in this collection. Plasmids pSCU-204-1 (88 kb) and pSCU-390-1 (90.7 kb), both from ST1193 (B2) isolates, are 99.9% identical in nucleotide sequence over the 88-kb length of pSCU-204-1 (Fig. 4). These plasmids are in turn closely related to the 90-kb plasmid pC32_1 from Shigella flexneri strain C32 and an 88-kb pNMEC-075A plasmid from E. coli ST1193 strain MCJCHV-1. Differences between all these plasmids are focused in a 20-kb variable region containing antibiotic resistance genes, shown in Fig. 4B.

## DISCUSSION

Only a small fraction of the thousands of E. coli genomes in the NCBI Genomes database are completely assembled. The work presented here was made possible by the development of affordable lab-scale long-read DNA sequencing (17, 18). This is essential for exploring the architecture of bacterial genomes, since chromosomes and plasmids are generally littered with repetitive transposable elements that preclude unambiguous assembly from short-read sequencing data. The goal of this study was to conclusively determine how antibiotic resistance genes are carried in commensal E. coli. To accomplish this, we generated 50 new, fully or near-fully assembled genomes using hybrid assemblers such as Unicycler and Flye (22, 23). As a caveat, we note that these assemblers employ distinct strategies that are affected differently by the quality and quantity of long- and short-read data (29). Flye has a higher residual error rate than Unicycler at the nucleotide level, so Unicycler assemblies were preferred for archiving in GenBank (37/50 chromosomes and nearly all plasmids from this project). However, when Flye was able to span gaps that Unicycler could not, the resulting assemblies were sufficient for the purposes of this project.

Previous population-based investigations of commensal E. coli plasmids and antibiotic resistance have relied on PCR to identify plasmid replicons (13, 30). These studies found replicon distributions similar to those reported here, with F replicons most abundant by far, followed by the I complex (B/O, K, Z, and I1). Whether particular replicon types were associated with higher frequencies of antibiotic resistance varied. Johnson et al. (30) found positive associations between FIA, FIA, and FIB replicons and several antibiotic resistance traits, and Marcadé et al. (31) found that *bla*_TEM-1_ genes are strongly associated with F replicons. On the other hand, Moran et al. (13) noted that only B/O replicons were significantly more abundant in antibiotic-resistant isolates. Using contemporary DNA sequencing methods, we determined that in the commensal E. coli we analyzed, 66% of acquired resistance genes were located on plasmids containing F replicons and 10% were on plasmids with I-complex replicons, compared to 19% residing on the chromosome.

For the most part, the large plasmids carrying antibiotic resistance genes were not highly conserved, perhaps due to their “cargo” (including antibiotic resistance genes) being in constant flux due to mobile elements. Plasmids pSCU-204-1 and pSCU-390-1from ST1193 isolates are intriguing exceptions, as their structures are very similar and align closely with a plasmid (pNMEC-075A) from the only other fully assembled ST1193 genome in GenBank (32), as well as with a plasmid (pC32_1) from a Shigella flexneri isolate. Johnson et al. (33) recently reported that, based on draft genome sequences, plasmids similar to pNMEC-075A are likely present in many E. coli ST1193 isolates. The ST1193 lineage is globally distributed, and it has emerged within the United States in the past decade as a significant extraintestinal pathogen (33). What key functions this conserved plasmid may provide to E. coli ST1193, other than serving as the primary platform for mobile antibiotic resistance genes, remain to be determined. Notably, this plasmid completely lacks the genes associated with the F-plasmid conjugal machinery, and yet its presence in a *Shigella* isolate suggests that it is still capable of horizontal transmission between cells.

Large, low-copy-number plasmids make up vastly less of the DNA content of E. coli cells than chromosomal DNA. Why most transposable elements carrying resistance genes are located on these small fractions of the genome is unknown. Tn*7* is one of the few transposons known to have a preferred integration site on the chromosome, but it nevertheless has a strong preference for insertion into conjugal plasmids (34). Sequence-independent factors related to replication mechanism (as in the case of Tn*7*), topology, or methylation state may influence target preference, and in turn may be influenced by host factors. Five of the 10 (50%) complete genomes we assembled from phylogroup D isolates contained acquired resistance genes on their chromosomes, a much higher frequency than the collection as a whole (6/29 isolates with acquired resistance genes [21%]). The types of plasmids, resistance genes, and mobile elements observed in phylogroup D isolates did not appear to be distinct from those in the remainder of isolates with acquired resistance genes, but perhaps as-yet unidentified host factors in this lineage influence the distribution of mobile elements between plasmids and chromosomes.

Very few transposable elements were observed on small mobilizable plasmids, despite their diversity and apparent abundance. Transposition onto small plasmids can occur; indeed, pSCU-105-2 likely resulted from transposition of a 6-kb Tn*2* (*bla*_TEM-1_) macrolide resistance module onto a ColE1 plasmid backbone. Numerous nearly identical homologs to pSCU-120-3 are found in GenBank, including p12579_4 from E. coli O55:H7 strain RM12579, an enteropathogenic strain isolated in California in 1974 (35), and pCERC2, identical in a commensal E. coli isolate from Australia in 2012 (36). The authors noted that these plasmids had likely been circulating globally in human-associated E. coli for decades, indicating their stability. Nevertheless, the low frequency of small plasmids carrying resistance genes in E. coli suggests that transpositional events involving small plasmids are generally inhibited, usually unstable, or are selected against. This may be fortunate for the human host, given that high-level expression of a resistance gene on a high-copy-number plasmid can potentiate a higher level (and in the case of β-lactams broader-spectrum) of antibiotic resistance (37, 38). The 9.7-kb pSCU-105-2 plasmid may illustrate this, as despite the plasmid-borne *bla*_TEM-1_ gene being wild type in sequence, SCU-105 displays an enhanced resistance to cephalosporins not seen in other isolates with this gene.

From the bacterial perspective, clustering of resistance genes on plasmids is advantageous for facilitating dramatic and simultaneous gains in resistance to multiple antibiotics. Nevertheless, the evolutionary dilemma of the “plasmid paradox” reflects the assumption that plasmid replication and maintenance costs exacted on the host are only offset under conditions where the plasmid explicitly provides a selective advantage, such as in the presence of antibiotics (39). Under such conditions, the plasmid is a symbiont; in their absence, the plasmid is a parasite. It should therefore be advantageous for resistance genes to move to the chromosome, where the host could benefit from them at a reduced cost. Recent experimental work on plasmid-host relationships (40) suggests that plasmid-host coevolution and compensatory mutations can reduce costs of plasmid maintenance and favor continued carriage of resistance genes and other genetic cargo on plasmid vectors. These findings have implications as well for the movement of such plasmids into new hosts (41); clearly there is much still to learn in this field.

Understanding the mobility of antibiotic resistance genes within genomes, within species, and within the microbiome at large can provide critical insights into trends in drug resistance among pathogens. The work presented here focuses on commensal E. coli, many of which can convert into opportunists causing extraintestinal infections (e.g., urinary tract infections [UTIs] or sepsis) (42). Almost half of the isolates examined here were from phylogroup B2, from which most extraintestinal pathogenic E. coli (ExPEC) strains derive (43, 44), and common ExPEC types represented among them included ST95 (12 isolates), ST1193 (4 isolates), ST73 (3 isolates), ST131 (3 isolates), and ST69 (phylogroup D, 8 isolates). The potential for F and other conjugal plasmids to facilitate acquisition of antibiotic resistance in E. coli and related species, including *Shigella*, *Klebsiella*, *Enterobacter*, *Salmonella*, and *Citrobacter*, will continue to be explored in future work.

## MATERIALS AND METHODS

### Strains and media.

Commensal E. coli bacteria were obtained from self-administered rectal swabs by study participants (college students aged 19 to 22 years old) over a 6-year period from 2014 to 2019. The study protocol and informed consent documents were approved by the Human Subjects Research Committee at Santa Clara University. Swabs were streaked on CHROMagar Orientation agar (CHROMagar, Paris, France) (45) containing no antibiotics and incubated at 37°C for 16 to 24 h. Colonies were identified by color and restreaked for isolation. No more than one isolate per student was included in the data reported here. Isolates were identified to the species level by the API20E system (bioMérieux) and/or 16S rRNA sequencing. Isolates used in this work are described in Tables S1 and S2 in the supplemental material.

### DNA sequencing, assembly, and analysis.

Genomic DNA was prepared from broth-grown cultures using the Macherey Nagel microbial DNA isolation kit. DNA preparations were assessed by agarose gel electrophoresis, UV spectroscopy, and Qubit fluorometry. Library preparation and sequencing with the Illumina MiSeq platform followed the manufacturer’s recommendations. 150-bp paired-end reads were trimmed based on length and quality using BBDUK (https://jgi.doe.gov/data-and-tools/bbtools/). *De novo* assembly of Illumina reads was done using the Geneious assembler (BioMatters LTD, Auckland, New Zealand). Long-read sequencing on the Oxford Nanopore MinION instrument followed the native genomic DNA barcoding sequencing protocol (protocol LSK108, Oxford Nanopore Technologies). MinION data were processed in MinKNOW (v. 3.6.5) using the Guppy basecaller (v.3.2.10), and demultiplexed by Epi2Me (Oxford Nanopore Technologies). Genome assemblies are described in Table S1 (isolates with completely or near-completely assembled genomes) and Table S2 (isolates with draft assemblies only). GenBank accession information is provided in Table 2 and Table S1; GenBank entries include metadata such as read coverage.

Assembly of MinION reads, combined with MiSeq reads, was done with the Unicycler (version 0.4.8) hybrid assembler (22). When genome assembly could not be achieved with Unicycler, Flye (version 2.6) (23) was applied to the same data. Following assembly with Flye, contigs were polished with Pilon (46) using short-read data. Unicycler assemblies were preferred, as Pilon polishing of Flye contigs leaves a significant residual error rate of 0.2 to 1%, but this did not interfere with the ultimate goal of localizing genes to plasmids or chromosomes.

Annotation was done by RAST v2 (47). Assembled genomes were analyzed using several tools from the Center for Genomic Epidemiology (https://cge.cbs.dtu.dk/services/), including ResFinder v3.2 (48) for identifying acquired antibiotic resistance genes and/or relevant mutations, MLST version 2.0 for multilocus sequence typing (49), and PlasmidFinder version 1.3 for identification of plasmid replicons (21). IncI complex plasmids were differentiated into B/O, I, K, and Z subtypes by comparison to the *repA* sequences for the respective subtypes recommended by Moran et al. (13). Other mobile genetic elements were identified using the Galileo Antimicrobial Resistance (GAMR) software (ArcBio, Cambridge, MA, USA), which is derived from the Multiple Antibiotic Resistance Annotator (MARA) database (24).

### Phenotypic testing.

Antimicrobial susceptibility testing was performed by Kirby-Bauer disk diffusion assays, using guidelines from the manufacturer (Hardy Diagnostics). Antibiotics tested included β-lactams (ampicillin and cephalothin), aminoglycosides (gentamicin, kanamycin, and streptomycin), chloramphenicol, quinolones (nalidixic acid and norfloxacin), macrolides (azithromycin), tetracyclines, sulfonamides (sulfamethoxazole), and trimethoprim.

### Analysis of plasmid mobilization of antibiotic resistance.

Plasmid DNA was isolated from E. coli cultures using the ZR Plasmid Miniprep Classic kit (Zymo Research) and analyzed on 1% agarose gels. Because large plasmids are not recovered with high efficiency from plasmid preparations, both plasmid and genomic DNA samples were used for electroporation with commercial electrocompetent E. coli NEB5α (New England Biolabs). Colonies were selected on LB agar plus ampicillin (50 μg/ml), streptomycin (50 μg/ml), gentamicin (20 μg/ml), or oxytetracycline (10 μg/ml).

### Data availability.

All complete or nearly complete E. coli genome sequences described herein have been archived in GenBank as part of BioProject PRJNA624897. Individual GenBank accession numbers are provided in Table 2 and Table S1.
