# Supplementary material for: F Plasmids Are the Major Carriers of Antibiotic Resistance Genes in Human-Associated Commensal Escherichia coli
Source: mSphere. 2020 Aug 5;5(4):e00709-20. doi: 10.1128/mSphere.00709-20 (PMC7407071; doi:10.1128/mSphere.00709-20)
Supplement: TABLE S1 [file mSphere.00709-20-st001.docx]

**Supplementary Table S1. Genome components for all commensal *E. coli* isolates completely or near-completely assembled in this work.**

| **Phylogroup** | **MLST**^a^ | **Isolate Number** | **Resistance Phenotype(s)**^b^ | **Assembly Type**^c^ | **Genome Component (GenBank Accession Number)** | **Size** | **Resistance Genes ^d,e^** | **Plasmid Replicon(s)**^f^ |
| --- | --- | --- | --- | --- | --- | --- | --- | --- |
| A | 10 | SCU-103 | AMP, CEF AZM NAL STR SXT TET | Flye | Chromosome  (CP054457) | 5.2 Mb | *gyrA* S83L | - |
|  |  |  |  | Uncicyler | pSCU-103-1  (CP054458) | 139 kb | *aadA5, dfrA17, bla_CTX-M-27_, mphA, strA, strB, sul1, sul2, tetA* | F1A, F1B, FII |
|  |  |  |  |  | pSCU-103-2  (CP054459-CP054460) | 83 kb^e^ | - | B/O/K/Z (Z) |
|  |  |  |  |  | pSCU-103-3  (CP054461) | 7.0 kb | - | - |
|  |  |  |  |  | pSCU-103-4  (CP054462) | 3.2 kb | - | - |
|  |  |  |  |  | pSCU-103-5  (CP054463) | 2.1 kb | - | Col(BS512) |
|  |  |  |  |  | pSCU-103-6  (CP054464) | 1.5 kb | - | Col(MG828) |
|  |  | SCU-107 | - | Unicycler | Chromosome  (CP053384) | 4.8 Mb | - | - |
|  |  |  |  |  | pSCU-107-1_SSU5-like phage  (CP053388) | 108 kb | - | - |
|  |  |  |  |  | pSCU-107-2  (CP053385) | 71 kb | - | FII |
|  |  |  |  |  | pSCU-107-3  (CP053386) | 5.2 kb | - | - |
|  |  |  |  |  | pSCU-107-4  (CP053387) | 2.1 kb | - | - |
|  |  | SCU-118 | AMP NAL STR  SXT TET | Unicycler | Chromosome  (CP051716) | 4.6 Mb | *gyrA* S83L | - |
|  |  |  |  |  | pSCU-118-1  (CP051717) | 85 kb | *aadA1, dfrA1, bla_TEM-1B_, mphB, strA, strB, sul1, sul2, tetA* | F1B' |
|  |  |  |  |  | pSCU-118-2  (CP051718) | 4.7 kb | - | - |
|  | 8125 | SCU-104 | NAL | Unicycler | Chromosome  (CP053284) | 5.2 Mb^e^ | *gyrA* S83L | - |
|  |  |  |  |  | pSCU-104-1  (CP053285) | 1.5 kb | - | Col(MG828) |
| B1 | 52 | SCU-152 | - | Unicycler | Chromosome  (CP051698) | 4.8 Mb | - | - |
|  |  |  |  |  | pSCU-152-1  (CP051699) | 117 kb | - | F1B', FII |
|  |  |  |  |  | pSCU-152-2  (pending) | 5.6 kb |  |  |
|  |  |  |  |  | pSCU-152-3  (pending) | 2.1 kb |  |  |
|  | 164 | SCU-478 | NAL | Unicycler | Chromosome  (CP054564.1, CP054563.1) | 5.2 Mb^e^ | *gyrA* S83L | - |
|  |  |  |  |  | pSCU-478-1  (CP054565.1) | 116 kb^e^ | - | F1C |
|  |  |  |  |  | pSCU-478-2  CP054566.1) | 5.3 kb | - | - |
|  | 3695 | SCU-106 | STR TET | Unicycler | Chromosome (CP053234) | 5.2 Mb^e^ | - | - |
|  |  |  |  |  | pSCU-106-1 (CP053235) | 159 kb | - | B/O/K/Z (Z) |
|  |  |  |  |  | pSCU-106-2 (CP053236) | 112 kb | *strA, strB, tetA* | F1B', F1C(II) |
|  |  |  |  |  | pSCU-106-3 (CP053237) | 11 kb | - | - |
|  |  |  |  |  | pSCU-106-4 (CP053238) | 8.1 kb | - | - |
|  |  |  |  |  | pSCU-106-5 (CP053239) | 7.6 kb | - | - |
|  |  |  |  |  | pSCU-106-6 (CP053240) | 5.7 kb | - | - |
|  |  |  |  |  | pSCU-106-7 (CP053241) | 5.2 kb | - | Col156 |
|  |  |  |  |  | pSCU-106-8 (CP053242) | 4.1 kb | - | - |
|  |  |  |  |  | pSCU-106-9 (CP053243) | 3.9 kb | - | Col(MP18) |
|  |  |  |  |  | pSCU-106-10 (CP053244) | 3.1 kb | - | - |
|  |  | SCU-308 | AMP STR SXT | Unicycler | Chromosome (CP053281) | 5.0 Mb | - | - |
|  |  |  |  |  | pSCU-308-1 (CP053282) | 152 kb | *bla_TEM-1B_, dfrA5, strA, strB, sul2* | F1B', FII |
|  |  |  |  |  | pSCU-308-2 (CP053283) | 68 kb | - | L/M' |
|  | 5974 | SCU-113 | - | Unicycler | Chromosome (CP051765) | 4.8 Mb | - | - |
|  | Unknown | SCU-483 | NAL | Flye | Chromosome  (CP054314) | 4.9 Mb^e^ | *gyrA* S83L | - |
|  |  |  |  |  | pSCU-483-1  (CP054315) | 89 kb | - | B/O/K/Z (K) |
|  |  |  |  |  | pSCU-483-2  (CP054316) | 80 kb | - | FII |
| B2 | 14 | SCU-387 | AMP AZM | Unicycler | Chromosome  (CP051688) | 5.3 Mb | - | - |
|  |  |  |  |  | pSCU-387-1  (CP051689) | 140 kb | - | F1B', FII |
|  |  |  |  |  | pSCU-387-2  (CP051690) | 39 kb^e^ | *bla_TEM-1B_,* *mphA* | FII |
|  |  |  |  |  | pSCU-387-3  (CP051691) | 6.2 kb | - | Col156 |
|  | 73 | SCU-112 | AMP, CEF (int) | Unicycler | Chromosome  (CP051725) | 5.1 Mb | - | - |
|  |  |  |  |  | pSCU-112-1  (CP051726) | 104 kb | *aadA1'*, *bla_SHV-1_* | F1B', FII, Col156 |
|  | 91 | SCU-121 | TET | Flye | Chromosome  (CP054328) | 5.0 Mb | - | - |
|  |  |  |  |  | pSCU-121-1  (CP054329) | 68 kb | *tetA* | FII |
|  |  |  |  |  | pSCU-121-2  (CP054330) | 9.1 kb | - | - |
|  |  |  |  |  | pSCU-121-3  (CP054331) | 5.6 kb | - | ColRNAI |
|  |  |  |  |  | pSCU-121-4  (CP054332) | 4.1 kb | - | Col(MG828) |
|  |  |  |  |  | pSCU-121-5  (CP054333) | 3.7 kb | - | Col156 |
|  |  |  |  |  | pSCU-121-6  (CP054334) | 1.5 kb | - | Col(MG828) |
|  | 95 | SCU-108 | AMP | Unicycler | Chromosome  (CP051735) | 5.1 Mb | - | - |
|  |  |  |  |  | pSCU-108-1  (CP051736) | 117 kb | - | F1B', FII, Col156 |
|  |  |  |  |  | pSCU-108-2  (CP051737) | 72 kb | *bla_TEM-1B_* | FII |
|  |  | SCU-116 | - | Unicycler | Chromosome  (CP051719) | 5.0 Mb | - | - |
|  |  |  |  |  | pSCU-116-1  (CP051720) | 84 kb |  | B/O/K/Z (Z) |
|  |  |  |  |  | pSCU-116-2  (CP051721) | 65 kb |  | F1A, F1B' |
|  |  |  |  |  | pSCU-116-3  (CP051722) | 5.2 kb |  | - |
|  |  |  |  |  | pSCU-116-4  (CP051723) | 4.1 kb |  | - |
|  |  |  |  |  | pSCU-116-5  (CP051724) | 1.5 kb |  | Col(MG828) |
|  |  | SCU-122 | - | Unicycler | Chromosome  (CP051714) | 4.9 Mb | - | - |
|  |  |  |  |  | pSCU-122-1  (CP051715) | 114 kb |  | F1B', FII, Col156 |
|  |  | SCU-123 | AMP STR SUL | Unicycler | Chromosome  (CP051711) | 5.0 Mb | - | - |
|  |  |  |  |  | pSCU-123-1  (CP051712) | 117 kb | - | F1B', FII, Col156 |
|  |  |  |  |  | pSCU-123-2  (CP051713) | 95 kb | *bla_TEM-1C_, strA, strB'*, *sul2* | B/O/K/Z (B/O) |
|  |  | SCU-306 | Azm Sul Tri | Unicycler | Chromosome (CP053231) | 5.0 Mb^e^ | - | - |
|  |  |  |  |  | pSCU-306-1 (CP053232) | 129 kb^e^ | *aadA2, dfrA12, mphA, sul1* | F1B, FII, Col156 |
|  |  |  |  |  | pSCU-306-2_SSU5-like phage (CP053233) | 112 kb | - | F1B |
|  |  | SCU-487 | - | Flye | Chromosome  (CP054454) | 5.0 Mb | - | - |
|  |  |  |  | Unicycler | pSCU-487-1  (CP054455) | 141 kb |  | F1B', FII |
|  |  |  |  |  | pSCU-487-2  (CP054456) | 4.1 kb |  | - |
|  |  | SCU-488 | - | Flye | Chromosome  (CP054449) | 5.1 Mb | - | - |
|  |  |  |  |  | pSCU-488-1  (CP054450) | 160 kb |  | F1B, FII, Col156 |
|  |  |  |  |  | pSCU-488-2  (CP054451) | 7.0 kb |  | - |
|  |  |  |  |  | pSCU-488-3  (CP054452) | 5.1 kb |  | Col156 |
|  |  |  |  |  | pSCU-488-4  (CP054453) | 1.6 kb |  | - |
|  | 131 | SCU-182 | AMP GEN NAL NOR | Unicycler | Chromosome  (CP054372) | 5.0 Mb^e^ | *gyrA* S83L D87N; *parC* S80I E84V | - |
|  |  |  |  |  | pSCU-182-1  (CP054376, CP054375, CP054374, CP054373) | 168 kb^e^ | *aac(3)-IId, bla_TEM-1B_* | F1A, F1B, FII, Col156 |
|  |  |  |  |  | pSCU-182-2  (CP054377) | 5.2 kb^e^ | - | - |
|  |  |  |  |  | pSCU-182-3  (CP054378) | 4.1 kb | - | - |
|  |  | SCU-481 | AMP, AMC AZM (INT) CHL SXT | Flye | Chromosome  (JABLYB010000000) | 4.9 Mb^e^ | - | - |
|  |  |  |  | Unicycler | pSCU-481-1  (JABLYB010000000) | 144 kb | *aadB, aacC', cmlA6, aadA5, dfrA17, sul1, bla_TEM-34_, mphA* | F1A, F1B, FII |
|  |  |  |  |  | pSCU-481-2_SSU5-like phage  (JABLYB010000000) | 108 kb | - | - |
|  |  |  |  |  | pSCU-481-3  (JABLYB010000000) | 4.1 kb | - | - |
|  |  |  |  |  | pSCU-481-4  (JABLYB010000000) | 2.3 kb | - | Col(MP18) |
|  | 144 | SCU-125 | STR  SXT | Unicycler | Chromosome  (CP051700) | 5.1 Mb | - | - |
|  |  |  |  |  | pSCU-125-1  (CP051701) | 121 kb | - | F1B', FII, Col156 |
|  |  |  |  |  | pSCU-125-2  (CP051702) | 93 kb | *dfrA5, strA, strB, sul1, sul2* | B/O/K/Z (Z) |
|  |  |  |  |  | pSCU-125-4  (CP051704) | 5.2 kb | - | - |
|  |  |  |  |  | pSCU-125-5  (CP051705) | 2.3 kb | - | - |
|  | 357 | SCU-124 | AMP | Unicycler | Chromosome  (CP051706) | 4.8 Mb | - | - |
|  |  |  |  |  | pSCU-124-1  (CP051707) | 114 kb | - | F1B', FII, Col156 |
|  |  |  |  |  | pSCU-124-2  (CP051708) | 73 kb | *bla_TEM-1B_* | FII |
|  |  |  |  |  | pSCU-124-3  (CP051709) | 12 kb^e^ | - | - |
|  |  |  |  |  | pSCU-124-4  (CP051710) | 1.5 kb | - | Col(MG828) |
|  | 420 | SCU-485 | - | Unicyler | Chromosome (CP053245) | 4.7 Mb^e^ | - | - |
|  |  |  |  |  | pSCU-485-1 (CP053246) | 112 kb | - | F1B', FII, Col156 |
|  | 491 | SCU-111 | - | Unicycler | Chromosome  (CP051727) | 4.8 Mb | - | - |
|  |  |  |  |  | pSCU-111-1  (CP051728) | 190 kb |  | F1A, F1C |
|  |  |  |  |  | pSCU-111-2  (CP051729) | 66 kb |  | FII |
|  |  |  |  |  | pSCU-111-3  (CP051730) | 6.8 kb |  | Col156 |
|  |  |  |  |  | pSCU-111-4  (CP051731) | 5.9 kb |  | - |
|  |  |  |  |  | pSCU-111-5  (CP051732) | 4.7 kb |  | - |
|  | 550 | SCU-176 | - | Unicycler | Chromosome  (CP054345) | 5.0 Mb | - | - |
|  |  |  |  |  | pSCU-176-1  (CP054346) | 39 kb^e^ |  | F1B, FII |
|  |  |  |  |  | pSCU-176-2  (CP054347) | 5.6 kb |  | - |
|  |  |  |  |  | pSCU-176-3  (CP054348) | 5.2 kb |  | - |
|  |  |  |  |  | pSCU-176-4  (CP054349) | 5.2 kb |  | Col156 |
|  |  |  |  |  | pSCU-176-5  (CP054350) | 4.1 kb |  | - |
|  |  |  |  |  | pSCU-176-6  (CP054351) | 3.9 kb |  | - |
|  |  |  |  |  | pSCU-176-7  (CP054352) | 1.8 kb |  | Col(MG828) |
|  | 657 | SCU-171 | - | Unicycler | Chromosome  (CP054363) | 5.2 Mb | - | - |
|  |  |  |  |  | pSCU-171-1  (CP054364) | 126 kb |  | F1B', FII |
|  |  |  |  |  | pSCU-171-2  (CP054365) | 31 kb |  | - |
|  |  |  |  |  | pSCU-171-3  (CP054366) | 8.9 kb |  | - |
|  |  |  |  |  | pSCU-171-4  (CP054367) | 3.1 kb |  | - |
|  | 998 | SCU-484 | NAL | Unicycler | Chromosome  (CP051744) | 5.1 Mb | *gyrA* S83L | - |
|  |  |  |  |  | pSCU-484-1  (CP051745) | 80 kb | - | I |
|  |  |  |  |  | pSCU-484-2  (CP051746) | 6.8 kb | - | Col156 |
|  |  |  |  |  | pSCU-484-3  (CP051747) | 6.3 kb | - | - |
|  |  |  |  |  | pSCU-484-4  (CP051748) | 4.1 kb | - | - |
|  | 1155 | SCU-115 | - | Unicycler | Chromosome  (CP054368) | 4.9 Mb | - | - |
|  |  |  |  |  | pSCU-115-1  (CP054369) | 148 kb |  | F1B', F1C |
|  |  |  |  |  | pSCU-115-2  (CP054370) | 34 kb |  | - |
|  | 1193 | SCU-147 | AMP AZM NAL NOR GEN STR SXT TET | Flye | Chromosome  (CP054325) | 5.1 Mb^e^ | *gyrA* S83L D87N; *parC* S80I | - |
|  |  |  |  |  | pSCU-147-1  (CP054326) | 105 kb | *aac(3)-IId, aadA5, dfrA17,* *bla_TEM-1B_*, *mphA, strA, strB, sul2, tetA* | F1A, F1B, Col156 |
|  |  |  |  |  | pSCU-147-2  (CP054327) | 4.1 kb | *-* | - |
|  |  | SCU-204 | NAL NOR STR SUL | Unicycler | Chromosome (CP053251) | 5.1 Mb | *gyrA* S83L D87N; *parC* S80I | - |
|  |  |  |  |  | pSCU-204-1  (CP054414.1) | 88 kb | *strA, strB, sul2* | F1A, F1B, Col156 |
|  |  |  |  |  | pSCU-204-2  (CP054413.1) | 87 kb | - | B/O/K/Z (I) |
|  |  |  |  |  | pSCU-204-3 _Unknown phage (CP053252) | 47 kb | - | - |
|  |  |  |  |  | pSCU-204-4 (CP053254) | 4.1 kb | - | - |
|  |  |  |  |  | pSCU-204-5 (CP053255) | 2.1 kb | - | Col(BS512) |
|  |  | SCU-390 | AMP NAL NOR STR SUL | Flye | Chromosome  (CP054319) | 5.0 Mb^e^ | *gyrA* S83L D87N; *parC* S80I | - |
|  |  |  |  |  | pSCU-390-1  (CP054321) | 91 kb | *bla_TEM-1B_, strA, strB,* *sul2* | F1A, F1B', Col156 |
|  |  |  |  |  | pSCU-390-2  (CP054320) | 42 kb | - | X1, X9 |
|  |  |  |  |  | pSCU-390-3  (CP054322) | 5.2 kb | - | Col156 |
|  |  |  |  |  | pSCU-390-4  (CP054323) | 4.1 kb | - | - |
|  |  |  |  |  | pSCU-390-5  (CP054324) | 2.1 kb | - | Col(BS512) |
|  | 1262 | SCU-101 | - | Unicycler | Chromosome  (CP051849) | 5.4 Mb | - | - |
|  |  |  |  |  | pSCU-101-1  (CP051850) | 7.5 kb |  | - |
|  |  |  |  |  | pSCU-101-2  (CP051851) | 5.0 kb |  | - |
|  |  |  |  |  | pSCU-101-3  (CP051852) | 1.0 kb |  | - |
|  | 2279 | SCU-479 | AMP, AMC, CEF CHL STR SUL TET | Flye | Chromosome  (CP054317) | 5.2 Mb | *bla_CTX-M-14_, bla_CMY-121_, bla_TEM-1B_, strA, strB, sul2, tetA* | - |
|  |  |  |  |  | pSCU-479-1  (CP054318) | 112 kb | - | F1B, FII, Col156 |
| D | 38 | SCU-164 | NAL SXT TET | Unicycler | Chromosome  (CP054343) | 5.4 Mb^e^ | *gyrA* S83L; *dfrA7, sul1, sul2, tetD* | - |
|  |  |  |  |  | pSCU-164-1  (CP054344) | 134 kb | - | F1B, FII, Col156 |
|  |  | SCU-397 | AMP, CEF CHL NAL STR SXT TET | Flye | Chromosome  (CP054828.1) | 5.4 Mb^e^ | *gyrA* S83L; *parC* S80I; *bla_CTX-M-14_* (2 copies)*, bla_TEM-1B_, catA1, dfrA7, strA, strB, sul1, sul2, tetD* | - |
|  |  |  |  | Unicycler | pSCU-397-1  (CP054829.1) | 144 kb^e^ | - | F1B, FII, Col156 |
|  |  | SCU-486 | AMP, CEF AZM GEN NAL STR SXT  TET | Unicycler | Chromosome  (CP051749) | 5.2 Mb | *gyrA* S83L; *bla_TEM-1B_, bla_CTX-M-14_, cat, strA, strB, sul2,* *tetD* | - |
|  |  |  |  |  | pSCU-486-1  (CP051750) | 84 kb | *aac(3)-IId, bla_CTX-M-14_, dfrA5, mphA,* *sul1* | F1B', FII |
|  | 69 | SCU-321 | - | Flye | Chromosome  (CP055158) | 4.7 Mb | - | - |
|  |  | SCU-313 | AMP AZM GEN NAL STR SXT TET | Unicycler | Chromosome  (CP051694) | 5.1 Mb | *gyrA* S83L | - |
|  |  |  |  |  | pSCU-313-1  (CP051695) | 105 kb | *aac(3)-IId, aadA5, dfrA17,* *bla_TEM-1B_, mphA, strA, strB, sul1, sul2, tetA* | F1A, F1B' |
|  |  |  |  |  | pSCU-313-2  (CP051696) | 67 kb | - | FII |
|  |  |  |  |  | pSCU-313-3  (CP051697) | 4.1 kb | - | - |
|  |  | SCU-482 | AMP AZM NAL STR SXT | Unicycler | Chromosome (CP053247) | 5.2 Mb^e^ | *gyrA* S83A | - |
|  |  |  |  |  | pSCU-482-1 (CP053248) | 145 kb | *aadA5, dfrA17, bla_TEM-1B_, mphA, strA, strB, sul1, sul2* | F1B', FII, Col156 |
|  |  |  |  |  | pSCU-482-2 (CP053249) | 5.2 kb | - | - |
|  |  |  |  |  | pSCU-482-3 (CP053250) | 1.5 kb | - | Col(MG828) |
|  | 106 | SCU-318 | AMP STR SUL TET | Flye | Chromosome  (CP051692) | 4.9 Mb | - | - |
|  |  |  |  |  | pSCU-318-1  (CP051693) | 105 kb | *bla_TEM-1B_, strA, strB*, *sul2,* *tetB* | F1B, FII |
|  | 394 | SCU-105 | AMP, CEF AZM STR SXT | Unicycler | Chromosome  (CP051738) | 5.2 Mb | *aadA1, dfrA1, sat2* | - |
|  |  |  |  |  | pSCU-105-1  (CP051739) | 173 kb^e^ | *strA, strB,* *sul2* | F1B, FII |
|  |  |  |  |  | pSCU-105-2  (CP051740) | 9.7 kb | *bla_TEM-1B_, mphA* | - |
|  |  |  |  |  | pSCU-105-3  (CP051741) | 7.9 kb | - | ColRNAI |
|  |  |  |  |  | pSCU-105-4  (CP051742) | 6.3 kb | - | - |
|  |  |  |  |  | pSCU-105-5  (CP051743) | 4.8 kb | - | - |
|  | 963 | SCU-109 | AMP, AMC, CEF GEN | Unicycler | Chromosome  (CP051733) | 5.0 Mb | *bla_CMY-2_* | - |
|  |  |  |  |  | pSCU-109-1  (CP051734) | 110 kb | *aac(3)-IId, bla_TEM-1B_* | F1B', FII, Col156 |
|  | 973 | SCU-102 | CEF (INT) | Unicycler | Chromosome  (CP051753) | 4.9 Mb | *ampC* attenuator mutation | - |
| E | 57 | SCU-316 | - | Unicycler | Chromosome  (CP054371) | 5.0 Mb | - | - |
| F | 62 | SCU-175 | AMP AZM STR  SXT TET | Unicycler | Chromosome  CP054379.1 | 5.4 Mb | - | - |
|  |  |  |  |  | pSCU-175-1  CP054380.1 | 124 kb | *aadA1, dfrA1, sat2, mphA, sul2, tetB* | B/O/K/Z (Z) |
|  |  |  |  |  | pSCU-175-2  CP054381.1 | 72 kb | *bla_TEM-1B_* | FII |
|  |  |  |  |  | pSCU-175-3  CP054382.1 | 51 kb | - | F1B', FII, Col156 |
|  |  |  |  |  | pSCU-175-4  CP054383.1 | 6.2 kb^e^ | - | - |
|  |  |  |  |  | pSCU-175-5  CP054384.1 | 5.2 kb | - | - |
|  |  |  |  |  | pSCU-175-6  CP054385.1 | 3.4 kb | - | - |
|  |  |  |  |  | pSCU-175-7  CP054386.1 | 1.8 kb | - | Col(MG828) |
|  | 67 | SCU-301 | - | Unicycler | Chromosome  (CP051751) | 5.1 Mb | - | - |
|  |  |  |  |  | pSCU-301-1  (CP051752) | 5.9 kb |  | - |
|  | 379 | SCU-172 | AMP | Unicycler | Chromosome  (CP054353) | 5.2 Mb | - | - |
|  |  |  |  |  | pSCU-172-1  (CP054354) | 83 kb | - | B/O/K/Z (B/O) |
|  |  |  |  |  | pSCU-172-2  (CP054355) | 79 kb | - | F1B', FII, Col156 |
|  |  |  |  |  | pSCU-172-3  (CP054356) | 76 kb | *bla_TEM-1B_* | FII |
|  |  |  |  |  | pSCU-172-4  (CP054357) | 5.7 kb | - | - |
|  |  |  |  |  | pSCU-172-5  (CP054358) | 5.2 kb | - | - |
|  |  |  |  |  | pSCU-172-6  (CP054359) | 4.1 kb | - | - |
|  |  |  |  |  | pSCU-172-7  (CP054360) | 2.3 kb | - | Col(MG828) |
|  |  |  |  |  | pSCU-172-8  (CP054361) | 2.1 kb | - | Col(BS512) |
|  |  |  |  |  | pSCU-172-9  (CP054362) | 1.6 kb | - | - |
|  | 648 | SCU-120 | AMP (INT), CEF AZM NAL NOR STR SXT TET | Flye | Chromosome  (CP054335) | 5.2 Mb | *gyrA* S83L D87N; *parC* S80I; *ampC* attenuator mutation | - |
|  |  |  |  |  | pSCU-120-1  (CP054336) | 143 kb | *aadA5, dfrA17, mphA, sul1, tetA* | F1A, F1B', FII |
|  |  |  |  |  | pSCU-120-2_P1-like phage  (CP054337) | 99 kb | - | - |
|  |  |  |  |  | pSCU-120-3  (CP054338) | 6.2 kb | *strA, strB, sul2* | - |
|  |  |  |  |  | pSCU-120-4  (CP054339) | 5.2 kb | - | Col156 |
|  |  |  |  |  | pSCU-120-5  (CP054340) | 4.8 kb | - | - |
|  |  |  |  |  | pSCU-120-6  (CP054341) | 2.1 kb | - | Col(BS512) |
|  |  |  |  |  | pSCU-120-7  (CP054342) | 1.6 kb | - | Col(MG828) |

^a^ Multi-locus sequence typing was done with the MLST algorithm through the Center for Genomic Epidemiology (<https://cge.cbs.dtu.dk/services/>) web portal.

^b^ Abbreviations: AMP = ampicillin; AMC = amoxicillin/clavulanic acid; AZM = azithromycin; CEF = cephalothin; CHL = chloramphenicol; GEN = gentamicin; KAN = kanamycin; NAL = nalidixic acid; NOR = norfloxacin; STR = streptomycin; SUL = sulfamethoxazole alone; SXT = sulfamethoxazole/trimethoprim; TET = tetracycline; TMP = trimethoprim alone. “Int” indicates that the size of the zone of inhibition for the antibiotic met the manufacturer’s criteria for “intermediate” resistance.

^c^ Assemblies were done with either Unicycler or Flye, using a combination of short- and long-read sequencing data. Flye assemblies were polished with short-read data using Pilon.

^d^*gyrA* and *parC* mutations that have previously been associated with quinolone and/or fluoroquinolone resistance are indicated with the amino acid residue that is changed, followed by the residue to which it has been changed.

^e^ Identification of antibiotic resistance genes was done with ResFinder. “ ' ” indicates that the identified antibiotic resistance gene was incomplete (between 60-90% present).

^f^ Identification of plasmid replicons was done with PlasmidFinder. “ ' ” indicates that the identified replicon sequence was incomplete (between 60-90% present).

^g^ Assembly was non-circular, suggesting gap of unknown size between ends.

**References**

1. Larsen MV, Cosentino S, Rasmussen S, Friis C, Hasman H, Marvig RL, Jelsbak L, Sicheritz-Pontén T, Ussery DW, Aarestrup FM, Lund O. 2012. Multilocus sequence typing of total-genome-sequenced bacteria. J Clin Micro 50:1355-61.

2. Wick RR, Judd LM, Gorrie CL, Holt KE. 2017. Unicycler: Resolving bacterial genome assemblies from short and long sequencing reads. PLoS Comput Biol 13(6): e1005595.

3. Kolmogorov M, Yuan J, Lin Y, Pevzner PA. 2019. Assembly of long, error-prone reads using repeat graphs. Nature Biotechnology. 37(5):540-6.

4. Walker BJ, Abeel T, Shea T, Priest M, Abouelliel A, Sakthikumar S, Cuomo CA, Zeng Q, Wortman J, Young SK, Earl AM. 2014. Pilon: an integrated tool for comprehensive microbial variant detection and genome assembly improvement. PloS One. 9(11).

5. Yoshida H, Bogaki MA, Nakamura MI, Nakamura SH. 1990. Quinolone resistance-determining region in the DNA gyrase *gyrA* gene of *Escherichia coli*. Antimicrob Agents Chemother 34(6):1271-2.

6. Heisig P. 1996. Genetic evidence for a role of *parC* mutations in development of high-level fluoroquinolone resistance in *Escherichia coli*. Antimicrob Agents Chemother 40(4):879-85.

7. Zankari E, Hasman H, Cosentino S, Vestergaard M, Rasmussen S, Lund O, Aarestrup FM, Larsen MV. 2012. Identification of acquired antimicrobial resistance genes. J Antimicrob Chemother 67: 2640–2644.

8. Carattoli A, Zankari E, Garcìa-Fernandez A, Larsen MV, Lund O, Villa L, Aarestrup FM, Hasman H. 2014. In silico detection and typing of plasmids using PlasmidFinder and plasmid multilocus sequence typing. Antimicrob Agents Chemother 58:3895-903.
