# Supplementary material for: F Plasmids Are the Major Carriers of Antibiotic Resistance Genes in Human-Associated Commensal Escherichia coli
Source: mSphere. 2020 Aug 5;5(4):e00709-20. doi: 10.1128/mSphere.00709-20 (PMC7407071; doi:10.1128/mSphere.00709-20)
Supplement: TABLE S2 [file mSphere.00709-20-st002.docx]

**Supplementary Table S2. Additional draft genome assemblies of commensal *E. coli* analyzed in this work.**

| **Phylogroup** | **MLST**^a^ | **Isolate Number** | **Draft Genome**  **Size** ^b^ | **Number of Contigs**^c^ | **Antibiotic Resistance Phenotype(s)**^d^ | **Resistance Genes or mutations ^e, f^** | **Plasmid Replicon(s) ^g^** |
| --- | --- | --- | --- | --- | --- | --- | --- |
| A | 10 | SCU-165 | 5.3 Mb | 138 | NAL | *gyrA* S83L | B/O/K/Z (Z) |
|  |  | SCU-186 | 5.0 Mb | 317 | NAL | *gyrA* S83L | B/O/K/Z (Z) |
|  |  | SCU-309 | 4.7 Mb | 23 | TET | *tetA* | F1B, F1C |
|  |  | SCU-400 | 4.6 Mb | 199 | - | - | - |
|  | 34 | SCU-319 | 5.0 Mb | 204 | AMP NAL STR SXT TET | *gyrA* S83A; *bla_TEM-1_, dfrA17, strA, strB, sul2, tetA* | B/O/K/Z (K), F1B, FII |
|  | 43 | SCU-399 | 4.5 Mb | 390 | AMP STR SUL | *bla_TEM-1_, strA, strB, sul2* | FII |
|  | 206 | SCU-389 | 5.0 Mb | 3 | NAL (INT) | - | F1B, FII |
|  | 216 | SCU-146 | 4.8 Mb | 109 | - | - | F1A, F1B |
|  | 685 | SCU-304 | 4.6 Mb | 155 | - | - | - |
|  |  | SCU-491 | 4.5 Mb | 216 | - | - | Col440I |
|  | 4381 | SCU-167 | 5.5 Mb | 132 | NAL SUL TET | *gyrA* S83L; *strA, strB, sul2, tetA* | B/O/K/Z (B/O), F1B, FII |
|  | Unknown | SCU-391 | 4.8 Mb | 261 | - | - | - |
| B1 | 155 | SCU-323 | 4.8 Mb | 125 | TET | *tetA* | F1B, F1C |
|  | 297 | SCU-127 | 4.9 Mb | 48 | - | - | F1B, FII |
|  |  | SCU-183 | 4.9 Mb | 195 | AMP CHL KAN SXT TET | *aadA2, aph(3')-Ia, bla_TEM-1_, cmlA1, dfrA12, sul2, tetB* | F1A, F1B, I |
|  | 641 | SCU-317 | 4.9 Mb | 171 | - | - | F1B, I |
|  | 847 | SCU-206 | 4.9 Mb | 56 | - | - | F1A, F1B, FII |
|  | 2163 | SCU-314 | 4.6 Mb | 147 | - | - | - |
|  | 2530 | SCU-324 | 4.8 Mb | 75 | - | - | - |
| B2 | 73 | SCU-322 | 4.9 Mb | 98 | - | - | - |
|  |  | SCU-388 | 5.0 Mb | 9 | - | - | F1B, FII, I |
|  | 95 | SCU-174 | 5.3 Mb | 97 | TET | *tetB* | F1A, F1B, FII |
|  |  | SCU-185 | 5.0 Mb | 372 | - | - | F1B, FII |
|  |  | SCU-305 | 5.0 Mb | 161 | - | - | F1B, FII |
|  |  | SCU-315 | 5.0 Mb | 81 | - | - | F1B, FII |
|  |  | SCU-488 | 5.3 Mb | 7 | - | - | F1B, FII, Col156 (2 copies), Col(MG828) |
|  | 127 | SCU-494 | 5.0 Mb | 192 | - | - | F1B, FII, Col156 |
|  | 131 | SCU-395 | 5.0 Mb | 99 | AMP, CEF AZM STR SXT TET | *aadA5, bla_CTX-M-27_, dfrA17, mphA, strA, strB, sul1, sul2, tetA* | F1B, FII, I |
|  | 141 | SCU-184 | 5.0 Mb | 327 | - | - | - |
|  | 429 | SCU-394 | 5.0 Mb | 107 | NAL STR SXT | *gyrA* S83L; *dfrA5, strA, strB, sul2* | F1A, FII |
|  | 452 | SCU-173 | 5.5 Mb | 185 | - | - | B/O/K/Z (Z) |
|  | 569 | SCU-398 | 4.7 Mb | 375 | AMP | *bla_TEM-1_* | FII |
|  | 1155 | SCU-169 | 5.4 Mb | 96 | - | - | F1B, F1C, FII (2 copies) |
|  | 1161 | SCU-320 | 5.0 Mb | 90 | - | - | F1B, FII |
|  | 1193 | SCU-490 | 4.8 Mb | 397 | - | - | I, Y |
|  | 1231 | SCU-188 | 5.0 Mb | 166 | AMP AZM STR SXT | *aadA2, dfrA12, bla_TEM-1_, mphA, sul1* | F1B, FII (2 copies) |
|  | 1459 | SCU-180 | 4.7 Mb | 48 | - | - | - |
|  | 4713 | SCU-153 | 5.0 Mb | 135 | AMP STR SXT | *bla_TEM-1_, dfrA5, strA, strB, sul2* | F1B, FII |
|  | 6091 | SCU-117 | 5.2 Mb | 106 | AMP STR (INT) SXT TET | *aadA1, bla_TEM-1_, dfrA1, sul1, tetB, tetD* | F1A, F1B, FII |
|  | Unknown | SCU-489 | 5.0 Mb | 397 | - | - | - |
| C | 88 | SCU-497 | 4.7 Mb | 234 | - | - | - |
| D | 69 | SCU-178 | 5.1 Mb | 389 | AMP AZM STR SXT TET | *aadA5, bla_TEM-1_, dfrA17, mphA, strA, strB, sul1, sul2, tetA* | F1B, FII |
|  |  | SCU-392 | 5.0 Mb | 263 | AZM GEN STR SUL TET | *aac(3)-IId, mphA, strA, strB, sul2, tetA* | F1A, F1B, FII |
|  |  | SCU-393 | 5.5 Mb | 302 | NAL STR (INT) SXT TET | *gyrA* S83L; *aadA5, dfrA17, sul1* (partial gene) *tetB* | F1A, F1B, FII |
|  |  | SCU-401 | 5.1 Mb | 315 | AMP AZM NAL (INT) GEN STR SXT TET | *aac(3)-IId, aadA5, bla_TEM-1_, dfrA17, mphA, qnrS1, strA, strB, sul1, sul2, tetA* | F1A, F1B, FII |
|  |  | SCU-477 | 5.4 Mb | 236 | AMP AZM CHL GEN NAL SXT TET | *gyrA* S83L; *aac(3)-IId, aadA5, bla_TEM-1_, dfrA17, mphA, sul1, tetA* | B/O/K/Z (Z), F1B, FII |
| E | 219 | SCU-493 | 4.8 Mb | 129 | - | - | - |
| F | 62 | SCU-114 | 4.8 Mb | 103 | - | - | F1B, FII (2 copies) |
|  | 379 | SCU-149 | 5.1 Mb | 203 | - | - | F1B, FII |
|  | 457 | SCU-166 | 5.0 Mb | 87 | - | - | - |
|  | 648 | SCU-128 | 5.0 Mb | 48 | AMP, CEF STR SXT TET | *bla_TEM-1_, dfrA8, strA, strB, sul2, tetA* | - |

^a^ Multi-locus sequence typing was done with the MLST algorithm through the Center for Genomic Epidemiology (<https://cge.cbs.dtu.dk/services/>) web portal.

^b^ Draft genome size was estimated from aggregation of all contigs in draft genome assembly.

^c^ Number of contigs greater than 1000 bp in length from *de novo* genome assembly.

^d^ Abbreviations: AMP = ampicillin; AMC = amoxicillin/clavulanic acid; AZM = azithromycin; CEF = cephalothin; CHL = chloramphenicol; GEN = gentamicin; KAN = kanamycin; NAL = nalidixic acid; NOR = norfloxacin; STR = streptomycin; SUL = sulfamethoxazole alone; SXT = sulfamethoxazole/trimethoprim; TET = tetracycline; TMP = trimethoprim alone. “Int” indicates that the size of the zone of inhibition for the antibiotic met the manufacturer’s criteria for “intermediate” resistance.

^e^ *gyrA* and *parC* mutations that have previously been associated with quinolone and/or fluoroquinolone resistance are indicated with the amino acid residue that is changed, followed by the residue to which it has been changed.

^f^ Identification of antibiotic resistance genes was done with ResFinder. “ ' ” indicates that the identified Ab^R^ gene was incomplete (between 60-90% present).

^g^ Identification of plasmid replicons was done with PlasmidFinder. “ ' ” indicates that the identified replicon sequence was incomplete (between 60-90% present).
